# Supplementary material for: Barriers and facilitators of severe acute malnutrition management at Felege Hiwot Comprehensive Specialized Hospital, Bahir Dar, North West Ethiopia, descriptive phenomenological study
Source: PLoS One. 2024 Mar 21;19(3):e0299575. doi: 10.1371/journal.pone.0299575 (PMC10956781; doi:10.1371/journal.pone.0299575)
Supplement: S1 File — (PDF) [file pone.0299575.s001.pdf]

Interview Questions for the study

Section I. Questions on socio-demographic characteristics of the study participants

**No**	**Questions**	**responses**	**skips**
101	Age of caregiver in years?<br><br>Sex of caregivers<br><br>Age of child<br><br>Sex of child		
102	Marital status?	1. Married<br><br>2. Single<br><br>3. Widowed<br><br>4. Divorced	
103	Educational status?	1. No formal education<br>2. Primary level (1-8)<br>3. Secondary level (9-12)<br>4. Tertiary level (12 and above)	
104	What is your ethnicity?	1. Amhara<br>2. Tigre<br>3. Oromo<br>4. Gurage<br>5. Other (specify)………	

105	What is your religion?	1. Orthodox<br>2. Muslim<br>3. Protestant<br>4. Catholic<br>5. Other (specify)……………	
106	What is your current occupation?	1. Farmer<br>2. Housewife<br>3. Employee<br>4. Merchant<br>5. Other specify……….	
107	How much is the monthly family income?	______ETB	
108	Where is your residence	1 Urban<br>2 Rural	
109	History of recurrent illness of the child, if yes state the health problems.	……………….	
110	Maternal health condition	1 Has no known illness<br>2 Has known illness<br>3 Other specify……….	
112	Immunization status of the child	1. Not vaccinated<br>2. Not fully vaccinated<br>3. Fully vaccinated<br>4. Other specify…….	
114	Place of delivery of this child	1.Home<br>2.Health post<br>3.Health center	

		4.Hospital<br>5.Other specify………	
115	Was there PNC follow up during delivering your child		
116	If yes for question 115, for how often?		
117	For how much you breast feed your child exclusively?		
118	Was there a history of SAM in other children?		
119	Was there any practice of GMP in your nearby facility?		
120	How many children do you<br>have?		

Section II. Interview guide for caregivers on barriers to SAM management

1. What is your role in the family?
2. What is the social situation and role?
3. How do you express severe malnutrition?
4. What are the signs of severe malnutrition?
5. What do you think are the causes of malnutrition?
6. What do you think about the risks and complications of malnutrition?
7. What impact would have on the child and family member as a result of malnutrition?
8. How do you and others in your community feel when your child gets severely malnourished?
9. Was there any malnourished child in your children before? If yes at what age and how it would be?
10. How do perceive the benefits and burden of treating SAM?

11. How do you describe the views and preferences in treating SAM at the household and community level? (What measures did you take? And why?
12. How do you come to this hospital? Who referred you?
13. What measures should be taken to prevent SAM?
14. How do you describe the use of substituent breast milk/formula milk? Have you ever used it? Why?

Section III. Interview guide for caregivers on facilitators of SAM management

1. What are your enablers/factors that make you adhere/seek health care in the management of SAM?
2. How do you describe the health service delivery at this hospital and other health facilities you visited? What things make you satisfied and what not?
3. How was the reception and description of services to you by health care providers?
4. How do you understand the overall layout of the hospital? Is it comfortable for you and your child? if not how?
5. How do you describe the availability of medicines and supplies to the management of SAM?
6. Are the health workers friendly and educate counsel, guide, and involve you in your child's care and treatment?
7. How do you describe the opportunities cascaded by the government of Ethiopia to enhance SAM management at all levels including you, health providers, and health managers?
8. How do you understand CBHI? Are you a member of CBHI or a pocket payer? How …? The impact of the cost expense to treat your child?
9. What measures need to be taken to strengthen the service by the government**?**

Section IV. Interview Guide for health care managers on SAM management

1 What do you think are the challenges /barriers/ obstacles of SAM management? Discuss it, please?

2 What do you think are the facilitators/enablers of SAM management? Discuss it please on behalf of your facility and others. (Free of charge /exempted? Capacity building, premises,)

3 What do you think are the opportunities for SAM management? Discuss it please (multi-sectoral support and collaboration, budget, supply, attention given by stakeholders including RHB and regional government and NGOs)

Section V. Interview guide for healthcare providers on SAM management

1 What do you think are the challenges /barriers/ obstacles of SAM management? Discuss it, please?

2 What do you think are the facilitators/enablers of SAM management? Discuss it please on behalf of your facility and others.

3 What do you think are the opportunities for SAM management? Discuss it, please

4 Would you explain the overall diagnostic and treatment protocols? Presence of guidelines and job aids?

Section VI. Observation guide for health care provider's practices and facility on SAM management

1 Observation of clinical practices and procedures, milk preparation, counseling, guidance and education, caregivers' involvement, bottle feeding? Whether according to the protocol for SAM management or not.

2 Observation of rooms and adequate ventilation, presence of washing facility (hand washing, shower for caregivers and child), check for play area and toys, painted rooms,

3 Observation of separated room for SAM management

4 Cleanliness of the service delivery area.

5 Observation of caregivers for hygiene, any special findings, and the condition of the child.

6 Observation of registers, and charts to ensure the data quality

7 Observe for cooking/food demonstration facility

8 Observe TV and job aids for health education.

9. Was emergency management at arrival of the hospital fast and courageous? Was there a problem?

10. Traditional Practices

11. Awareness creation, health education at the periphery/living area?
