# Supplementary material for: Barriers and facilitators of severe acute malnutrition management at Felege Hiwot Comprehensive Specialized Hospital, Bahir Dar, North West Ethiopia, descriptive phenomenological study
Source: PLoS One. 2024 Mar 21;19(3):e0299575. doi: 10.1371/journal.pone.0299575 (PMC10956781; doi:10.1371/journal.pone.0299575)
Supplement: Attachment — Submitted filename: Response to Reviewers.docx [file pone.0299575.s002.docx]

**Date: October 25, 2023**

**Rebuttal letter**

PONE-D-23-21702

Barriers and facilitators of severe acute malnutrition management at Felege Hiwot Comprehensive Specialized Hospital, North West Ethiopia, Bahir Dar, a descriptive phenomenological study

**Zeamanuel Anteneh Yigzaw**

**To Plos one**

**Dear all,**

We the authors of this manuscript are pleased to thank the journal editors and the reviewers for revising the manuscript and giving your valuable and constructive comments and suggestions that help to improve the manuscript. We have made a rigorous revision of the manuscript as per your questions and comments. We have included the point-by-point response in the table below framed as editors’ comments/questions and authors’ responses. **We have made a severe revision on the entire manuscript that we believe had merit in improving the manuscript and attached it as tracked change and clean version separately**. We are happy to receive additional revisions if any that would have merit in improving the manuscript.

| **Journal Requirements** | **Authors Response** |
| --- | --- |
| 1. Please ensure that your manuscript meets PLOS ONE's style requirements, including those for file naming. The PLOS ONE style templates can be found at <https://journals.plos.org/plosone/s/file?id=wjVg/PLOSOne_formatting_sample_main_body.pdf> and  <https://journals.plos.org/plosone/s/file?id=ba62/PLOSOne_formatting_sample_title_authors_affiliations.pdf> . | **Thank you very much**  We prepare it based on the journal template |
| 2. Thank you for stating the following financial disclosure:  "No funding at all" | **Thank you again**  We rewrite as per your comment as “The authors received no specific funding for this work’’. |
| **3** Thank you for stating the following in your Competing Interests section:   "No authors have competing interests" | **Thank you again**  We rewrite as per your comment as “The authors have declared that no competing interests exist”. |
| **4.** We note that you have indicated that data from this study are available upon request. PLOS only allows data to be available upon request if there are legal or ethical restrictions on sharing data publicly. For more information on unacceptable data access restrictions, please see <http://journals.plos.org/plosone/s/data-availability#loc-unacceptable-data-access-restrictions>. | **Thank you again**  All relevant data are within the manuscript and its supporting information |
| In your revised cover letter, please address the following prompts:  a) If there are ethical or legal restrictions on sharing a de-identified data set, please explain them in detail (e.g., data contain potentially sensitive information, data are owned by a third-party organization, etc.) and who has imposed them (e.g., an ethics committee). Please also provide contact information for a data access committee, ethics committee, or other institutional body to which data requests may be sent. | **Thank you for your feedback**  Our data does not contain potentially sensitive information |
| b) If there are no restrictions, please upload the minimal anonymized data set necessary to replicate your study findings as either Supporting Information files or to a stable, public repository and provide us with the relevant URLs, DOIs, or accession numbers. For a list of acceptable repositories, please see <http://journals.plos.org/plosone/s/data-availability#loc-recommended-repositories>.  We will update your Data Availability statement on your behalf to reflect the information you provide. | **Thank you again**  We upload the English Version Interview guide as a supporting information |
| 5. Your ethics statement should only appear in the Methods section of your manuscript. If your ethics statement is written in any section besides the Methods, please move it to the Methods section and delete it from any other section. Please ensure that your ethics statement is included in your manuscript, as the ethics statement entered into the online submission form will not be published alongside your manuscript.  [Note: HTML markup is below. Please do not edit.] | **Thank you again**  We incorporate the ethics letter in the methods section. |
| **Reviewer Comments**  **Comments to the author** | **Author’s Response** |
| 1. Is the manuscript technically sound, and do the data support the conclusions?  The manuscript must describe a technically sound piece of scientific research with data that supports the conclusions. Experiments must have been conducted rigorously, with appropriate controls, replication, and sample sizes. The conclusions must be drawn appropriately based on the data presented. | **Thank you again for your critique.**  Yes. The manuscript was technically sound and the data support the conclusion.  Since the manuscript is done with exploratory research with a phenomenology approach the conclusion supports the data. |
| 2. Has the statistical analysis been performed appropriately and rigorously? | **Thank You again**  Yes. The statistical analysis was performed appropriately and rigorously. |
| 3. Have the authors made all data underlying the findings in their manuscript fully available? | **Thank you very much again!**  Yes  The authors made all data underlying the findings |
| 4. Is the manuscript presented in an intelligible fashion and written in standard English? | **Thank you again**  The manuscript is presented and written in standard English. |
| **5. Reviewer Comments to the author** | **Author’s Response** |
| Reviewer #1: The research area is very alarming. However, choosing the study area is not in line with the current problem of the country and the region. I recommend including rural areas as well as urban areas for a better understanding of barriers and facilitators of severe acute malnutrition management. | **Thank you for your feedback**  We think that the study area was appropriate because the study area catchment population includes both urban and rural areas especially rural areas, it covers over 5 million people in the region. |
| Why chose exploratory Phenomenology qualitative research? | **Thank you again**  Because there are different types of qualitative research these are explanatory and exploratory. since we follow pure qualitative research, we do prefer exploratory phenomenology. To study in an in-depth way.  In addition, exploratory research is used to understand a relatively unknown problem or phenomenon, particularly in cases where little is understood about the topic. |
| How do you come up with samples (9,3,2)? | **Thank you again**  we select 9 caregivers or mothers, 3 health care workers, and 2 health care managers.  We triangulate it to get different perspectives from caregivers or mothers, healthcare workers, and healthcare managers.  Since they are different, they have different perspectives therefore since it is qualitative research and since it is one issue om |
| Why does the PI only do the thematic analysis? How did you control bias? | **Thank you again**  The PI did the thematic analysis because in qualitative research the PI was the main researcher.  In addition, most of the time qualitative research analysis is done with data collection. Therefore, in qualitative research, the PI is the data collector.  We control bias by using alternative explanations, reviewing findings, and verifying the data using observation, |
| Reviewer #2:   1. The rigor of the study is indicated Through triangulations i.e., Data source triangulation and method triangulation (observation and qualitative). In order to make the work free of bias, the author did not seem to triangulate via investigators' triangulation as well. | **Thank you again.**  Since it is pure qualitative research, the PI does all the things required. We use triangulation using both observation and qualitative interviews. |
| 2. The full name of the Atlas-Ti software, the year, and the exact version number should be mentioned | **Thank You**  Atlas Ti Version 7 software, 2016 |
| 3. How did service providers recognize the 2 types of SAM, while this could be a facilitator/barrier for the treatment? | **Thank you again**  Service providers recognize the two types of SAM because since they are professionals, they know the clinical manifestation. |
| References are not checked and corrected such as the Author's surname, Article title, Journal Name, Volume (Issue), and pp. page range. It seems the author relied on citation software without cross-edition. For e.g:2,5,7,11,17,23,34 and more | **Thank you again**  We cross-edit based on your comments therefore we reamend or reinsert the references that are not found in Google Scholar and PubMed (now it is a neat and clear nice document). Now the document has 31 references found in Google Scholar. |
