# Supplementary material for: Barriers and facilitators of severe acute malnutrition management at Felege Hiwot Comprehensive Specialized Hospital, Bahir Dar, North West Ethiopia, descriptive phenomenological study
Source: PLoS One. 2024 Mar 21;19(3):e0299575. doi: 10.1371/journal.pone.0299575 (PMC10956781; doi:10.1371/journal.pone.0299575)
Supplement: Attachment — Submitted filename: Response to Reviewers.docx [file pone.0299575.s003.docx]

**Date: January 9, 2024**

**Rebuttal Letter**

PONE-D-23-21702R1

Barriers and facilitators of severe acute malnutrition management at Felege Hiwot Comprehensive Specialized Hospital, North West Ethiopia, Bahir Dar, a descriptive phenomenological study

PLOS ONE

**Zeamanuel Anteneh Yigzaw**

**To Plos one**

**Dear all,**

We the authors of this manuscript are pleased to thank the journal editors and the reviewers for revising the manuscript and giving your valuable and constructive comments and suggestions that help to improve the manuscript. We have made a rigorous revision of the manuscript as per your questions and comments. We have included the point-by-point response in the table below framed as editors’ comments/questions and authors’ responses. **We have made a severe revision on the entire manuscript that we believe had merit in improving the manuscript and attached it as tracked change and clean version separately**. We are happy to receive additional revisions if any that would have merit in improving the manuscript.

| **Reviewers' comments:  Reviewer's Responses to Questions**  Comments to the Author | **Authors Response** |
| --- | --- |
| 1. If the authors have adequately addressed your comments raised in a previous round of review and you feel that this manuscript is now acceptable for publication, you may indicate that here to bypass the “Comments to the Author” section, enter your conflict-of-interest statement in the “Confidential to Editor” section, and submit your "Accept" recommendation. | **Thank you very much** |
| 2. Is the manuscript technically sound, and does the data support the conclusions?  The manuscript must describe a technically sound piece of scientific research with data that supports the conclusions. Experiments must have been conducted rigorously, with appropriate controls, replication, and sample sizes. The conclusions must be drawn appropriately based on the data presented. | **yes** |
| 3. Has the statistical analysis been performed appropriately and rigorously? | **yes** |
| 4. Have the authors made all data underlying the findings in their manuscript fully available? | **yes** |
| 5. Is the manuscript presented in an intelligible fashion and written in standard English? | **Yes** |
| **Review Comments to the Author** |  |
| Reviewer #3: |  |
| - The rigor of the study is very weak and questionable as researchers based their results on mere self-reported data from 15 participants. This is a big issue in this study since the validity and credibility of results are questionable and not convincing. | Thank you very much  Trustworthiness or rigor of a study refers to the degree of confidence in data, interpretation, and methods used to ensure the quality of a study. We add some sentences to strengthen the trustworthiness of the study.  See lines 168 – 196 in the track change |
| - Using the phenomenological design is unclear and requires further justifications | Thank you very much  This design is used to attain the lived experience and an understanding of the right meaning of a phenomenon of interest through engaging detailed descriptions of the mothers or caregivers and service providers’ perceptions toward barriers and facilitators while managing severe acute malnutrition.  phenomenological research studies lived experiences to gain deeper insights into how people understand those experiences.  Phenomenological research design is descriptive. The researcher aims to describe as accurately as possible the structure of a phenomenon.  Phenomenological research design focuses on exploring the essence of human experiences and understanding the meaning people attribute to those experiences. It seeks to capture the underlying structures and core aspects of these experiences without imposing preconceived theories or interpretations.  We put a reference see line 120 – 124 in the track change |
| - Sampling is not convincing and needs more justifications | Thank you very much  Creswell suggests that a reasonable sample size may range from 3 – 25 participants for phenomenological study design. In addition, in qualitative research, it depends on the level of saturation.  Therefore, the final sample size taken was fifteen (15), nine care providers, three healthcare managers, and three healthcare providers. We use in-depth interviews for caregivers and key informant interviews for HCWs and HCMs.  See lines 142 – 151 in the track change |
| - Themes are vague and cannot reflect the results | Thank you very much  The study has two major themes and six sub-themes.  We prepare a table to clear themes and subthemes see table 2 or line 224 |
| - Conclusions cannot drawn from the study | Thank you very much  We rewrote it again as per your comment and added some paragraphs. see lines 633 – 640 in the track change. |
| - Background is very weak and needs to be strengthened in light of international studies | Thank you very much  We add some paragraphs and add some statements as per your comment see lines 53 – 108. |
| - You should generate the research gap for this study | Thank you very much  We try to address the research gap as a strength and limitation of the study and we rewrite it again as per your comment. See lines 610 – 620 in the track change. For example, since the study was phenomenological study design Researcher-induced bias can affect the outcome of the study. But we try to reduce it. |
| - You should follow the guidelines of scientific writing | Thank you very much  We followed the Plos one qualitative guideline to write this manuscript, therefore based on your comment, I avoided the operational definition part because mostly it was common in quantitative studies. See lines 201 – 206 in the track change. |
| - Discussion does not follow guidelines of effective debate. It requires in-depth analysis and more criticism | Thank you very much  We rewrite it again as per your comment. And we add some paragraphs as per your comment. See line 493 - 609. We add lines 511,518, 523, 544, and 573 in the track change. We removed from lines 597 – 609 in the track change. |
| - What about the implications of this study? How this study could add to clinicians and health care management. How this study could shape health policy in Ethiopia? You should add a separate section for implications | Thank you very much  We add the implications of the study as per your comment see lines 622 – 629 in the track change. |
| - The majority of references cited are outdated (more than five years) | Thank you very much  We add more than Ten (10) recent references that are not longer than five years of study as per the research protocol. See lines 684 – 809 in the Track Change |

Thank you very much
