# Supplementary material for: Barriers and facilitators of severe acute malnutrition management at Felege Hiwot Comprehensive Specialized Hospital, Bahir Dar, North West Ethiopia, descriptive phenomenological study
Source: PLoS One. 2024 Mar 21;19(3):e0299575. doi: 10.1371/journal.pone.0299575 (PMC10956781; doi:10.1371/journal.pone.0299575)
Supplement: Attachment — Submitted filename: Response to Reviewers.docx [file pone.0299575.s004.docx]

**Date: January 31, 2024**

**Rebuttal Letter**

PONE-D-23-21702R2

Barriers and facilitators of severe acute malnutrition management at Felege Hiwot Comprehensive Specialized Hospital, North West Ethiopia, Bahir Dar, a descriptive phenomenological study

PLOS ONE

**Zeamanuel Anteneh Yigzaw**

**To Plos one**

**Dear all,**

We the authors of this manuscript are pleased to thank the journal editors and the reviewers for revising the manuscript and giving your valuable and constructive comments and suggestions that help to improve the manuscript. We have made a rigorous revision of the manuscript as per your questions and comments. We have included the point-by-point response in the table below framed as editors’ comments/questions and authors’ responses. **We have made a severe revision on the entire manuscript that we believe had merit in improving the manuscript and attached it as tracked change and clean version separately**. We are happy to receive additional revisions if any that would have merit in improving the manuscript.

| **Comments to the Author** | **Authors Response** |
| --- | --- |
| 2. Is the manuscript technically sound, and does the data support the conclusions? | **Thank you very much**  The manuscript is technically sound and is generally well-written. The manuscript describes a technically sound piece of scientific research with data that supports the conclusions. Experiments must have been conducted rigorously, with appropriate controls, replication, and sample sizes. |
| 3. Has the statistical analysis been performed appropriately and rigorously? | **Thank you very much**  Yes, the statistical analysis was performed appropriately and rigorously. |
| 4. Have the authors made all data underlying the findings in their manuscript fully available? | **Thank you very much**  Yes, all relevant data are within the manuscript and its supporting information file. |
| 5. Is the manuscript presented in an intelligible fashion and written in standard English? | **Thank you very much**  Yes, they were presented in standard English and an intelligible fashion |
| **Review Comments to the Author** |  |
| Reviewer #3: |  |
| The conclusion cannot be drawn from this study. | **Thank you very much**  The conclusion from this study was we think that clear that the barriers and facilitators of SAM were a multiplicity of factors starting from socio-cultural factors up to service provision and others too.  We try to keep the coherence of the write-up. |
| - Data regarding rigor, sampling, and design are still not convincing at this point. The issue of reaching data saturation from the self-reporting of 15 subjects is very questionable | The rigor of the study was rewritten by keeping coherence again, To the best of our knowledge we try to address all the methods that were important for the trustworthiness of the study, including other literature. See lines 159 – 181.  **Regarding sampling: -** the sample size was 15, we think that it was enough because the issue is malnutrition, therefore the study aimed to explore the detailed lived experiences of the caregivers. In addition, know the experiences of health care workers for malnutrition children and the faces of caregivers and know the manager's overview about malnutrition. Therefore, since the topic is one, the 15 subjects were very enough to understand the phenomenon and to explore the issue raised malnutrition.  **Regarding design: -**  We use descriptive phenomenological study design because it describes the lived experiences of the caregivers and we put so many references or similar research done using this study design as evidence.  You can see lines 110 and 134. |
| - Theoretical underpinnings of the study are still weak | **Thank you very much**  We use phenomenological study design. Therefore, we use it as a foundation or as underpinning because phenomenology qualitative research gives a brief description of the lived experiences of the caregivers. |
| - Results are vague and need to be re-written in a clear and informative manner | **Thank you very much**  We try to write it in a good manner as per your comment by rephrasing some themes and sentences. |
| - Discussion of findings is not adequately explained | **Thank you very much**  We add discussions of the findings based on your recommendation see line 499 up to line 506 in the track change. More we added two references, and now the paper has 45 references, which adds value to the paper also. |

**Thank you very much**
